# Methyl jasmonate alleviates Cd-induced lipid peroxidation in spinach by enhancing photosynthesis and the antioxidant defence system

**DOI:** 10.1038/s41598-024-60950-6

**Published:** 2025-11-18

**Authors:** Mingjun Miao, Jiajia Li, Xiaokui Lei, Jichao Liao, Jian Zhong, Ju Li, Zhi Li, Liang Yang, Yanqin Ma, Yuejian Li, Wei Chang

**Affiliations:** 1 Horticulture Research Institute, Sichuan Academy of Agricultural Sciences, Vegetable Germplasm Innovation and Variety Improvement Key Laboratory of Sichuan Province, Chengdu, 610066 China; 2 Horticultural Crops Biology and Germplasm Enhancement in Southwest Regions Key Laboratory of Ministry of Agriculture and Rural Affairs, Chengdu, 610066 China; 3 Institute of Sichuan Edible Fungi, Chengdu, 610066 China; 4 Sichuan Vegetable Engineering Technology Research Center, Chengdu, 610066 China

**Keywords:** Cd stress, Growth physiology, Mitigation effects, Photosynthesis, Spinach, Agroecology, Environmental sciences, Environmental impact

## Abstract

Cadmium (Cd) is a potentially harmful element that adversely affects plant growth, physiology and biochemical metabolism. In the present study, we used hydroponics with foliar spraying with the hormone MeJA to explore the mitigating effects and possible mechanisms of methyl jasmonate (MeJA) on Cd toxicity in spinach. The effects of different concentrations of MeJA (1, 5, 25 and 100 μmol/L) on growth parameters, photosynthetic characteristics, physiological functions and Cd uptake and partitioning in spinach under Cd stress (50 µmol/L) were analysed. Compared with Cd treatment, exogenous supplementation with 25 μmol/L MeJA significantly increased spinach plant height by 58.30%, root length by 58.20%, stem thickness by 58.25% and aboveground biomass by 64.94%, while reducing the Cd content of the whole spinach plant by 24.56%. Exogenous application of MeJA increased photosynthesis by increasing the net photosynthetic rate (Pn), stomatal conductance (Gs), intercellular CO_2_ concentration (Ci), and transpiration rate (Tr), which decreased the stomatal limiting value (Ls) of spinach, resulting in a 15.72–65.78% increase in the chlorophyll content and a 20.98–60.23% increase in the carotenoids content. Moreover, MeJA mitigated reactive oxygen species (ROS) production by increasing peroxidase (POD), superoxide dismutase (SOD) and catalase (CAT) activity in response to oxidative stress, which in turn reduced the malondialdehyde (MDA) content, proline content and soluble protein content in Cd-stressed spinach plants, thereby improving the stress tolerance of spinach seedlings. Therefore, the results of the present study contribute to the understanding of the mechanism by which MeJA alleviates Cd toxicity in spinach.

## Introduction

Cadmium (Cd) is a highly toxic heavy metal element that can enter the human body through soils and plants, posing a serious threat to human health^1^. Contamination of agricultural soils as well as the environment has occurred at a rapid rate with the development of industry and increased urbanization, and contamination caused by the metal Cd has been rapidly increasing in agricultural and facility soils^2,3^. Globally, up to 30,000 tons of Cd are released into the environment every year^4^, and the total amount of Cd discharged into the environment from industrial waste in China is approximately 680 tons per year^5^. The Cd mass fraction in vegetables exceeded the standard by as much as 23.5%, up to 17-fold higher than the food hygiene standard^6^. Cd is readily absorbed by the plant root system and transferred to the aboveground parts, thus affecting plant growth and many physiological activities^7,8^. Therefore, the high mobility and toxicity of Cd make it imperative to develop effective and environmentally friendly strategies to control the harmful effects of Cd on plants.

An increase in Cd levels leads to its entry and accumulation in plants, which causes damage to plant physiology and growth. It is well known that the effects of Cd on plants cause disruption of chloroplast structure, a decrease in photosynthesis^9^, inhibition of transpiration, and ultimately a reduction in plant biomass and yield^10^. Cd stress leads to an imbalance in the metabolism of plant cells and induces the production of ROS, which cause oxidative damage in plants by inducing lipid peroxidation and affecting other biomolecules in the cell^11^. Moreover, Cd stress reduces the uptake and transport of nutrients (e.g., calcium (Ca), magnesium (Mg), iron (Fe), and Zn), leading to nutrient deficiencies and plant death^12^. Khan et al. showed that 50 μmol/L Cd significantly reduced biomass and decreased leaf photosynthetic parameters in *Brassica rapa* ssp. *chinensis* L^13^. As a result, finding ways or means to mitigate the damage caused by Cd stress to plants has become a critical issue in agricultural development.

Phytohormones are important components for improving plant resistance, signalling adverse conditions and stimulating plant adaptation pathways. MeJA, a derivative of jasmonic acid (JA), is a natural plant hormone involved in many biological processes^14,15^. It has been previously shown that MeJA increases plant resistance by regulating the antioxidant defence system in sugarcane seedlings^16^. Wang et al. reported that in okra, exogenous of MeJA enhances plant tolerance to Cd stress by regulating endogenous hormone metabolism, osmoregulatory substances, photosynthetic pigments, and ROS metabolism^17^. Additionally, MeJA enhances plant tolerance to heavy metal stress by regulating the expression of relevant genes and the production of secondary metabolites^18,19^. In conclusion, MeJA is involved in a variety of physiological and biochemical processes in plants, affecting seed germination, metabolic regulation, and defence responses^16,20,21^.

Spinach (*Spinacia oleracea* L.) is an edible annual flowering vegetable of the quinoa family (Chenopodiaceae) that is rich in essential vitamins, dietary fibre, minerals, and phytochemicals^22,23^. It is considered a healthy vegetable in the human diet and is commonly grown and consumed worldwide. It has the potential to absorb large amounts of heavy metals and toxic elements from inter roots and transport them to the edible parts^24^, and spinach leaves contain up to 367.7 mg/kg Cd^25^. According to the national “Soil environmental quality—Risk Control Standard for Soil contamination of development lands (Trial)” (GB 15618-2018, GB 36600-2018) and combined with the results of previous research^26,27^, the experimental Cd^2+^ concentration is 50 μmol/L. Most studies clearly show research on exogenously applied MeJA under Cd stress in crops such as pepper^21^, okra^17^, and rice^28^ have progressed from the phenotypic to the molecular level. However, there are few studies on the physiological mechanisms involved in the interaction between Cd and MeJA in spinach. Therefore, the present study aimed to investigate (1) the effects of different concentrations of MeJA on the growth and phenotype of spinach plants under Cd toxicity, (2) whether MeJA alleviates Cd toxicity by increasing the photosynthetic efficiency of spinach, and (3) whether MeJA spraying modulates antioxidant enzymes, membrane lipid peroxidation, and osmoregulatory substances, thereby reducing or eliminating the damage caused by Cd to spinach. The results of the present study will deepen the understanding of the phenomenon of Cd toxicity mitigation by exogenous MeJA, and will provide a means to develop strategies for mitigating drug damage in vegetable crops in Cd-contaminated areas.

## Materials and methods

### Materials for testing

The spinach variety “Greenway TY771” was obtained from Teng Yun Seed Trading Co. in Shijiazhuang, Hebei Province, China. The variety has a dark green leaf colour, is peach shaped, has spreading leaves, is an upright spinach, has low Cd absorption and has high disease resistance. This experiment was conducted at the College of Horticulture, Sichuan Agricultural University (Chengdu, China). The Cd compound was CdCl_2_·2.5H_2_O (analytically pure) and the test nutrient solution was Hoagland nutrient solution. Exogenously applied MeJA was purchased from Sigma, USA.

### Plant growth under different treatment regimens

Spinach seeds of uniform size, full grain and free from pests and diseases were uniformly placed in Petri dishes lined with wet filter paper at ambient temperature, protected from light and kept well hydrated. Germination was carried out in an artificial incubator at day and night temperatures of 20 °C/18 °C. Seeds were sown in cavity trays containing clean perlite and vermiculite (1:1 mix by volume) after 0.5 cm of dew. According to the growth conditions, the seedlings were sprayed with 1/4 Hoagland nutrient solution into the trays at the right time and in the right amount after emergence and allowed to grow in an artificial incubator at a temperature of 20 °C/18 °C (day/night) and a photoperiod of 12 h/12 h (day/night).

When the 4 true leaves of the seedlings were fully expanded, the strong and healthy seedlings (1 plant per pot) were transplanted into a 10 cm × 10 cm (bottom × height) nutrient bowl with perlite (the bottom of the bowl was padded with gauze to prevent the perlite from leaking out which played a role in fixation). After the seedlings had six leaves and one heart, the spinach leaves were sprayed with different concentrations of MeJA (1, 5, 25, 100 μmol/L) solution. After 3 d of pretreatment, the nutrient bowl was placed in plastic trays with a height of 8 cm, which were filled with Hoagland nutrient solution containing Cd at concentrations of 0 and 50 μmol/L (added in the form of CdCl_2_·2.5H_2_O). At the same time, different concentrations of MeJA solution were sprayed on the leaves at approximately 18:00. The plants were sprayed every 3 d for a total of 3 sprays to the point where the leaves dripped. The nutrient solution was replaced every 3 d.

**Figure 1:**
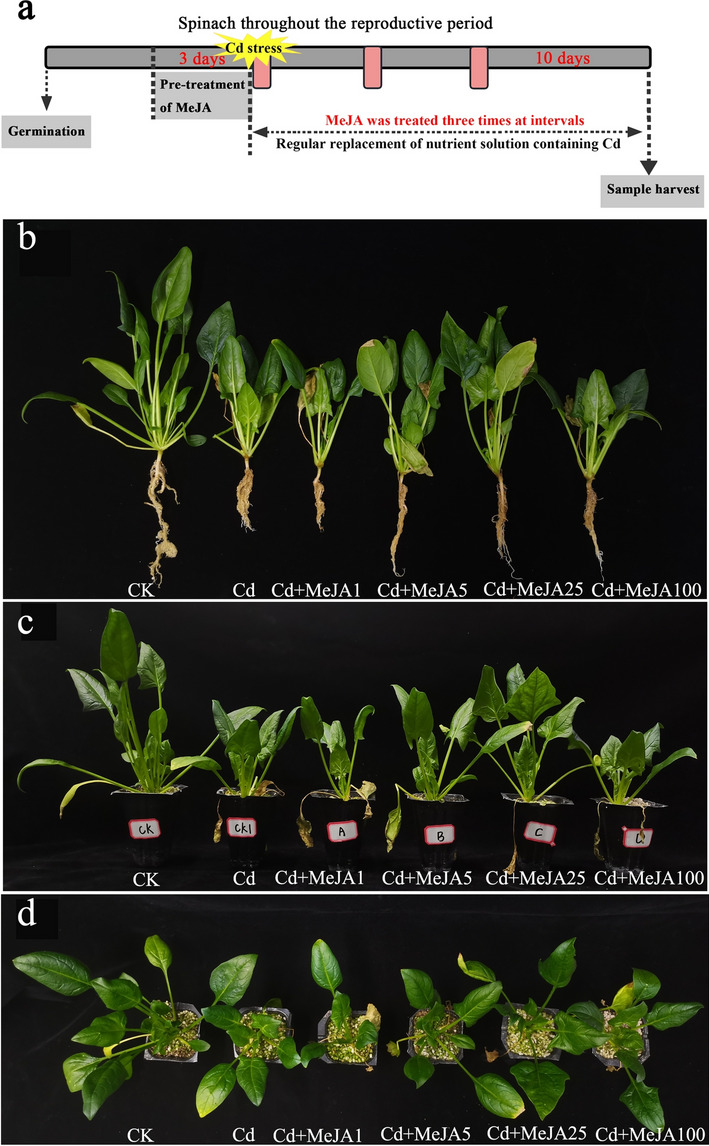
Phenotypic changes in spinach exogenously sprayed with MeJA under Cd stress. Note: (**A**) Scheme of the treatment used for exogenous spraying with MeJA on spinach after Cd stress, (**B**) frontal phenotypic view, (**C**) tray frontal view, (**D**) tray top view.

Throughout the experiment, MeJA solutions with different concentrations were sprayed four times (once time for pretreatment and three times for posttreatment with Cd). At least 30% of the nutrient solution was ensured to flows out when replacing the nutrient solution to prevent Cd from accumulating in the nutrient bowl. Cultivation with full nutrient solution without added Cd and water spraying was used as a blank control (CK), and cultivation with nutrient solution containing 50 μmol/L Cd and water spraying was used as a Cd treatment control. A total of 6 treatments (CK, Cd, Cd + MeJA1, Cd + MeJA5, Cd + MeJA25, and Cd + MeJA100) were replicated 3 times. The different treatments are outlined in followed Supplementary Table S1, and each treatment was replicated three times with four pots replicate. Plate-to-pan positions were exchanged at irregular intervals throughout the growth process to attenuate marginal effects and to control pests and diseases in a timely manner. Spinach was sampled at maturity 10 d after the last MeJA spray (Fig. 1A). After the whole plant was measured for growth and morphological indexes, fresh and dry samples were collected from the aboveground parts (edible parts) and roots. After sampling with liquid nitrogen, the fresh samples were stored in an ultralow-temperature refrigerator at − 80 °C for the determination of physiological indexes. The dry samples were heated in an oven at 105 °C for 15 min, dried at 75 °C until constant weight, and then pulverized for the determination of Cd content.

### Measurement methods

#### Measurement of growth index and biomass

Plant height and root length were measured using a millimetre scale. The plant samples were washed whole with tap water, rinsed repeatedly with ultrapure water and swabbed dry. The spinach was divided into aboveground parts (leaves, stems) and roots, and the fresh weights were weighed separately using an electronic balance. The aboveground parts (leaves, stems) and roots were subsequently divided. The plants were heated in an oven at 105 °C for 15 min, dried at 75 °C until constant weight, and weighed to determine the dry weight and biomass.

#### Quantification of photosynthetic parameters and photosynthetic pigments

The 2nd-3rd functional leaves of spinach were selected, and the Pn, Gs, Tr, Ci, and Ls of the spinach leaves were determined using a portable photosynthesis system LI-6400XT (LI-COR, USA). The endogenous light intensity was set at 1000 μmol/(m^2^-s), the CO_2_ concentration was 400 µl/L, and the temperature was 25 °C for the measure. Ls = 1 − Ci/Ca, where Ci/Ca is the ratio of the intercellular CO_2_ concentration to the ambient CO_2_ concentration. The Chlorophyll content and carotenoid content were determined by extraction with an acetone-ethanol mixture.

#### Determination of cell membrane permeability and osmoregulatory substances

The 2nd-3rd true leaves of each treated plant were selected, and mixed well, and the soluble sugar content was determined by anthrone colorimetry^29^; the soluble protein content was determined by the Coomassie brilliant blue method^30^; the proline content was determined by the sulfosalicylic acid method^31^; and the MDA content was determined by the thiobarbituric acid method^32^. Cell membrane permeability was determined by the conductivity method: 0.2 g of the spinach sample was weighed into 20 mL of deionized water, and shaken at ambient temperature for 30 min to determine the solution conductivity (A); then, the spinach sample was boiled at 100 °C for 20 min together with deionized water, and the solution conductivity was determined by adjusting to the preheating volume using deionized water (B). Relative conductivity (%) = conductivity (A)/conductivity (B) × 100%.

#### Analysis of antioxidant enzyme activities

The 2nd-3rd true leaves of each treatment plant were selected and mixed for the determination of the following indices. Superoxide dismutase (SOD) activity was determined by the nitrogen blue tetrazolium (NBT) method^33^, and its activity was determined by inhibiting the photoreduction of NBT by 50% as one unit of vigour (U). POD activity was determined by the guaiacol method^33^, and its activity was based on the change in A470 by 0.1 per minute as one unit of enzyme activity. CAT activity was determined by the UV-absorption method^34^, and its activity was based on 1 μmol of H_2_O_2_ decomposed in 1 min as one enzyme activity unit.

#### Determination of Cd content

The Cd content of the sample was determined via the microwave digestion method in GB5009.15-2014 Determination of Cd in Food. The dry sample (0.5 g, accurate to 0.0001 g) was weighed in a microwave digestion tank, and 5 mL of HNO_3_ and 2 mL of H_2_O_2_ were added. After digestion and cooling, the tank was opened, and the digestive solution was colourless or yellowish. The acid was heated to nearly dry, the tank was rinsed with a small amount of nitric acid solution (1%) 3 times, and the solution was transferred to a 10 mL volumetric flask containing nitric acid solution (1%), which was fixed to the scale, mixed and allowed to stand. The Cd content in 20 μL was determined by an atomic absorption spectrophotometer.

### Data statistics and analysis

Excel was used for statistical analysis, SPSS 21.0 was used for analysis of variance (ANOVA), Duncan’s multiple test was used for comparison of means, and analysis of the significance of differences in each trait measurement was performed. We used Origin 2021 to construct the graphs, and the data in the graphs are the means of three replicates.

1 $${\text{Cd transfer coefficient }}\left( {{\text{TF}}} \right) = {\text{shoot content}}/{\text{root content}}$$

2 $${\text{Membership function value}}:\mu \left( {X_{i} } \right) = \frac{{\left( {X_{i} - {\rm X}_{imin} } \right)}}{{\left( {X_{imax} - X_{imin} } \right)}},\;(i = {1},{ 2}, \ldots ,{\text{ n}})$$

3 $${\text{Comprehensive index value}}:F\left( {Xj} \right) = a_{1j} X_{1j} + a_{2j} X_{2j} + \cdots + a_{ij} X_{ij} ,\;(i = {1},{ 2}, \ldots ,{\text{ n;}}\;{\text{j}} = {1},{ 2}, \ldots ,{\text{n}})$$

4 $${\text{Comprehensive indicator weight}}:w_{j} = \frac{{P_{j} }}{{\mathop \sum \nolimits_{j = 1}^{n} P_{j} }},\;\left( {{\text{j}} = {1},{ 2}, \ldots ,{\text{n}}} \right)$$

5 $${\text{Comprehensive index evaluation}}:D = \mathop \sum \limits_{j = 1}^{n} \left[ {F\left( {X_{j} } \right) \times W_{j} } \right],\;\left( {{\text{j}} = {1},{ 2}, \ldots ,{\text{n}}} \right)$$

In the formulas, ${X}_{i}$ is the $i$-th comprehensive index. ${X}_{min}$ is the minimum value of the i-th comprehensive index, and ${X}_{max}$ is the maximum value of the i-th comprehensive index. For the j-th comprehensive index value of F(Xj), a_ij_ represents the eigenvector corresponding to the eigenvalues of each single index, and X_ij_ is the normalized value of each single index. In the formula, ${w}_{j}$ represents the weight of the jth comprehensive index in all comprehensive indexes, and ${P}_{j}$ denotes the variance contribution of the composite indicator of the jth of each treatment. D is the comprehensive evaluation value.

### Ethical approval

This study complied with the IUCN Policy Statement on Research Involving Species at Risk of Extinction and the Convention on the Trade in Endangered Species of Wild Fauna and Flora.

## Results

### Phenotypic and growth indexes of spinach performance

Spinach samples were collected at the maturity stage 10 d after the last application of MeJA. Cd stress caused obvious chlorosis symptoms on spinach leaves (Fig. 1B). Figure 1 shows that different concentrations of MeJA caused different degrees of improvement at the morphological level in spinach after Cd stress. In particular, the external morphology of spinach plants sprayed with MeJA25 improved greatly after Cd stress treatment (Fig. 1C). There were different levels of significance in the growth indexes of spinach after Cd stress caused by different concentrations of MeJA (Table 1, *p* < 0.05). Compared with those after Cd stress, plant height, root length, stem thickness, aboveground fresh weight, aboveground dry weight, belowground fresh weight and belowground dry weight significantly increased by 58.21%, 58.20%, 58.18%, 65.70%, 64.22%, 76.40% and 99.54%, respectively, under MeJA25 spray treatment conditions.

**Table 1:** Effects of spraying different concentrations of MeJA on the growth indexes of spinach under Cd stress conditions.

Treatment	Plant height (cm/plant)	Root length (cm/plant)	Stem thickness (mm/plant)	Aboveground fresh weight (g/plant)	Aboveground dry weight (g/plant)	Belowground fresh weight (g/plant)	Belowground dry weight (g/plant)
CK	20.63 ± 0.38a	19.90 ± 0.32a	13.72 ± 0.20a	16.89 ± 0.20a	1.39 ± 0.02a	2.64 ± 0.10a	0.17 ± 0.00a
Cd	10.37 ± 0.30d	10.37 ± 0.30d	9.40 ± 0.28d	9.07 ± 0.51d	0.77 ± 0.01e	0.89 ± 0.01d	0.07 ± 0.00e
Cd + MeJA1	10.27 ± 0.35d	10.93 ± 0.50d	9.90 ± 0.50d	9.78 ± 0.24d	0.93 ± 0.03d	0.85 ± 0.03d	0.07 ± 0.00e
Cd + MeJA5	13.13 ± 0.23c	12.80 ± 0.38c	11.84 ± 0.19c	11.93 ± 0.14c	1.04 ± 0.06c	1.18 ± 0.05c	0.10 ± 0.00d
Cd + MeJA25	16.40 ± 0.17b	16.40 ± 0.17b	14.86 ± 0.09b	15.02 ± 0.28b	1.27 ± 0.03b	1.57 ± 0.06b	0.14 ± 0.00b
Cd + MeJA100	12.70 ± 0.35c	13.83 ± 0.26c	12.50 ± 0.36c	11.94 ± 0.17c	0.97 ± 0.02 cd	1.30 ± 0.02c	0.12 ± 0.00c

Different lowercase letters indicate significant differences (*p* < 0.05) among treatments.

### Effect of different concentrations of MeJA on the photosynthetic characteristics of spinach

**Figure 2:**
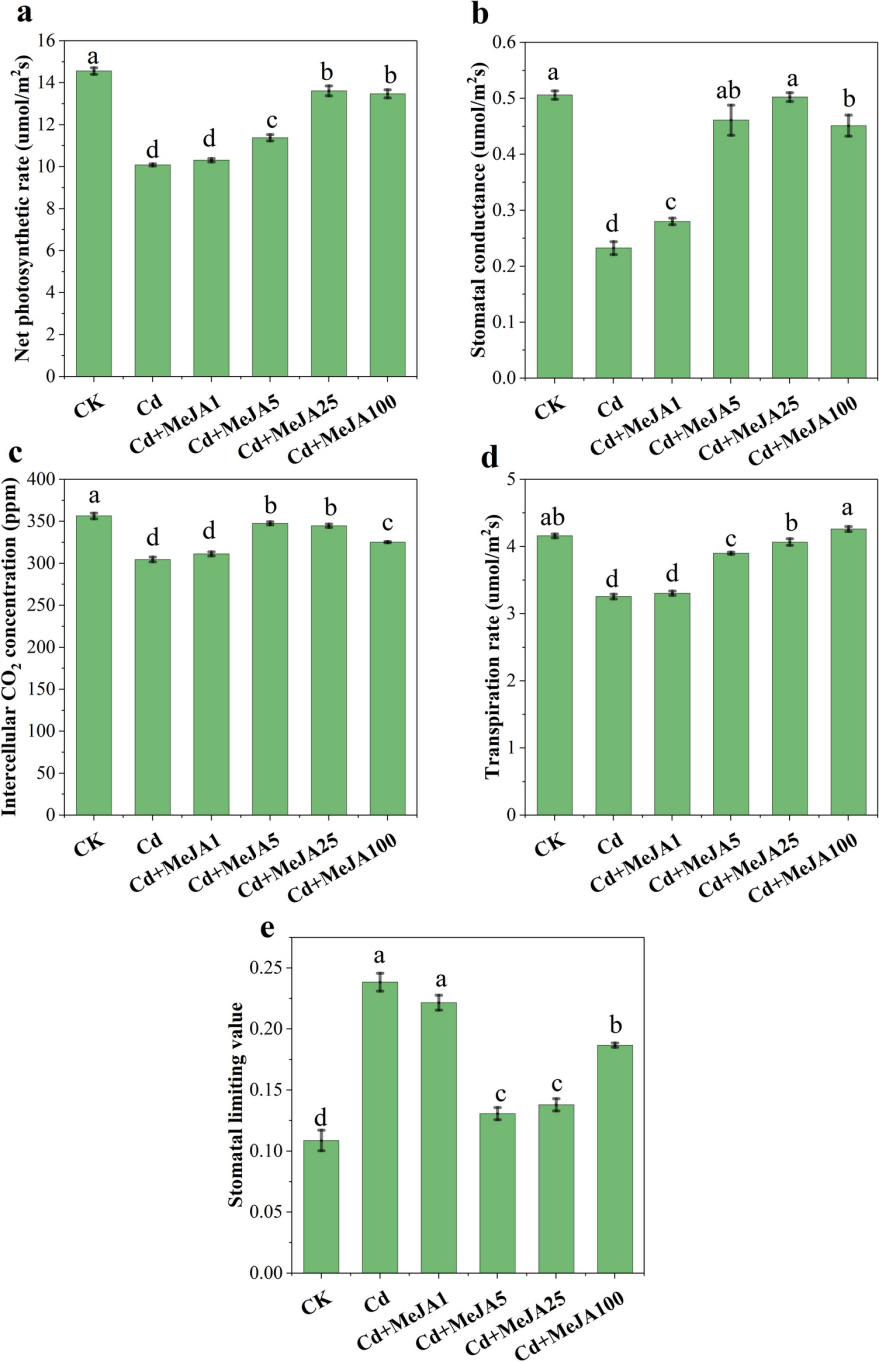
Apparent photosynthetic index of spinach leaves treated with different concentrations of MeJA. Note: (**A**) Net photosynthetic rate (Pn), (**B**) stomatal conductance (Gs), (**C**) intercellular CO_2_ concentration (Ci), (**D**) transpiration rate (Tr), and (E) stomatal limiting value (Ls). Different lowercase letters indicate significant differences (*p* < 0.05) among treatments, as follows.

The Pn, Gs, Ci, Tr, and Ls of spinach under different concentrations of MeJA were determined. As shown in Fig. 2, different concentrations of MeJA produced different degrees of mitigation effects on Cd stress. Compared with those under CK, spinach under Cd stress exhibited significant decreases in Pn, Gs, Ci, and Tr (Fig. 2A–D, *p* < 0.05) and a significant increase in Ls (Fig. 2E, *p* < 0.05). Under Cd stress, after MeJA5, MeJA25, and MeJA100 spraying, the Pn (12.79%, 34.99%, and 33.56%), Gs (98.29%, 116.09%, and 94.07%), Ci (14.14%, 13.20%, and 6.78%), and Tr (19.80%, 24.90%, and 30.87%) increased; respectively, and the Ls decreased (45.16%, 37.76%, and 42.85%). These results indicated that MeJA25 and MeJA100 spraying provided different degrees of relief after Cd stress. Therefore, gas exchange was significantly reduced under Cd stress, and exogenous spraying of MeJA restored Cd-induced injury.

### Changes in chloroplast pigments in spinach

**Figure 3:**
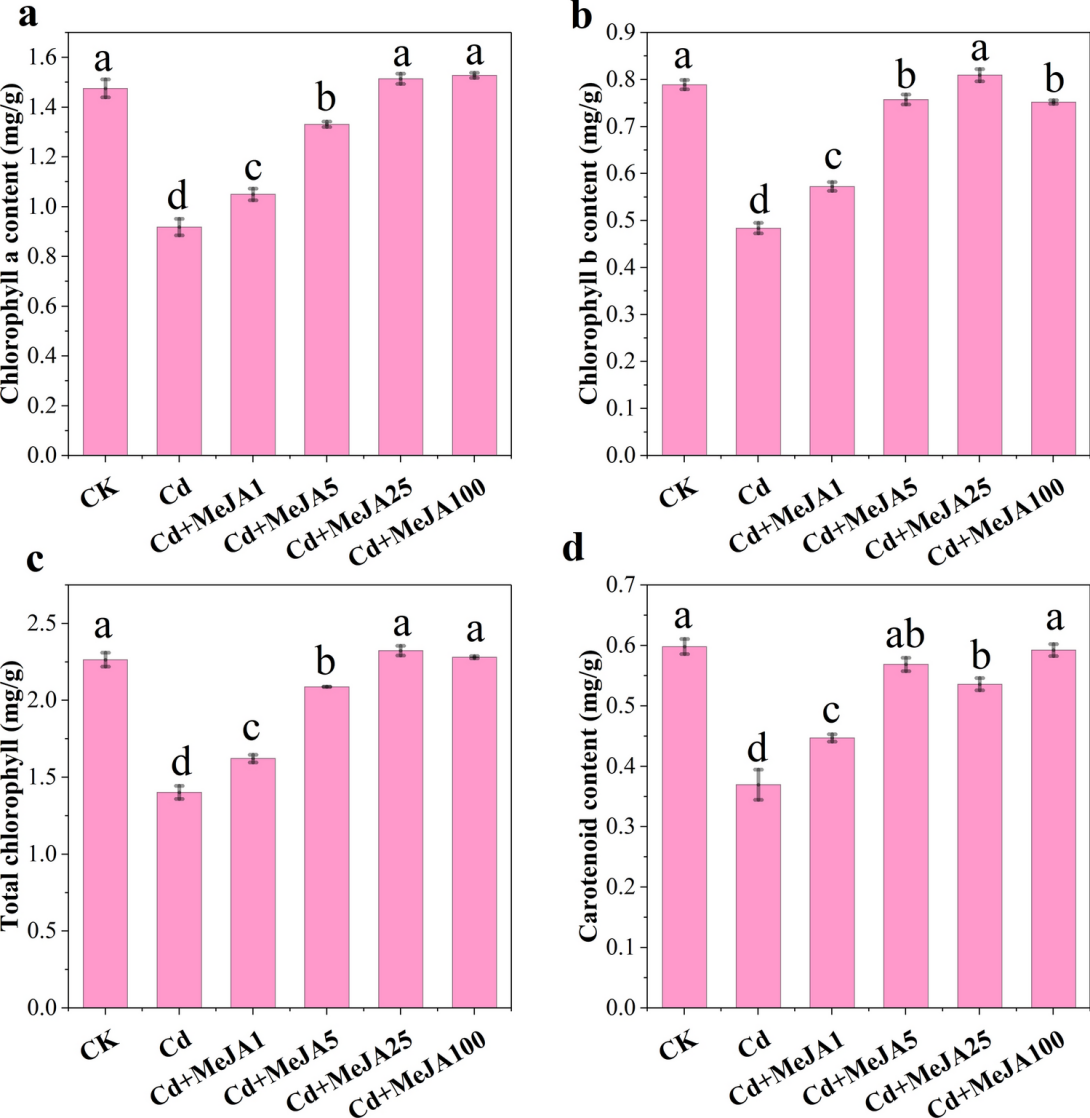
Changes in the chlorophyll content of spinach plants treated with different concentrations of MeJA. Note: (**A**) Chlorophyll a content, (**B**) chlorophyll b content, (**C**) chlorophyll content and (**D**) carotenoid content.

Compared with those in CK, significant decreases in chlorophyll a, chlorophyll b, chlorophyll and carotenoids were observed in spinach under Cd stress (Fig. 3A–D, *p* < 0.05). Under Cd stress, the supplementation with 25 μmol/L MeJA effectively increased the *chl b* and total *chl* contents by 67.24% and 65.78%, respectively, reaching the maximum values. The *chl a* and carotenoid contents increased (reached the maximum values) by 66.49% and 60.23%, respectively, after MeJA100 spraying. These results indicated that under Cd stress, different concentrations of MeJA had different degrees of relief on the photosynthetic pigments of spinach.

### Effects of different concentrations of MeJA on spinach membrane lipid peroxidation and osmoregulatory substances

**Figure 4:**
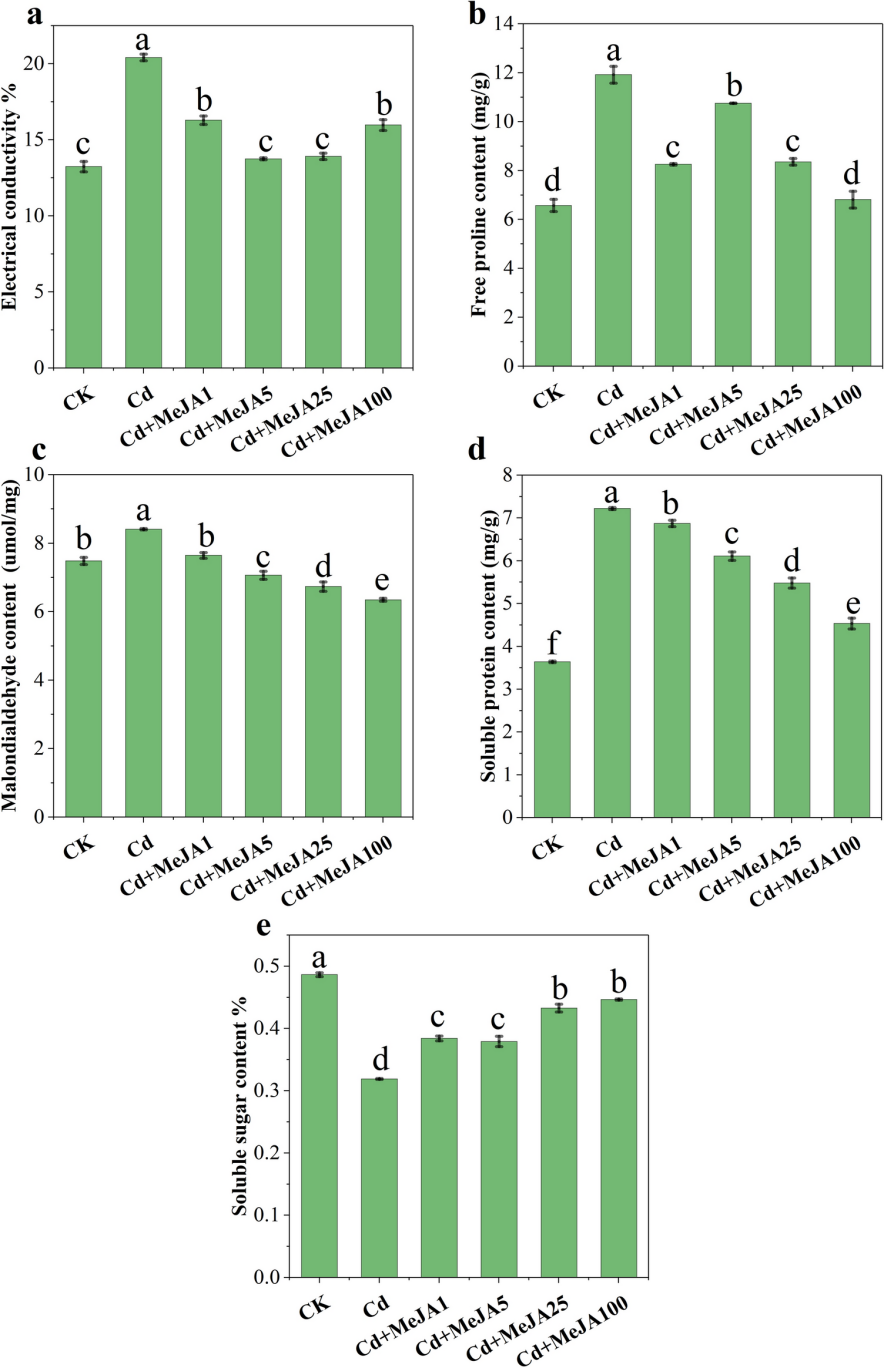
Membrane lipid peroxidation and osmoregulatory substances in spinach as affected by different concentrations of MeJA. Note: (**A**) electrical conductivity (%), (**B**) free proline content, (**C**) malondialdehyde (MDA) content, (**D**) soluble protein content and (E) soluble sugar content.

Under Cd stress, different concentrations of MeJA significantly affected the electrical conductivity, free proline content, MDA content, soluble protein content, and soluble sugar content (Fig. 4, *p* < 0.05). This was especially evident after MeJA5, MeJA25, and MeJA100 spraying, where the respective electrical conductivity (32.61%, 31.83%, and 21.75%), free proline content (9.80%, 29.90%, and 42.86%), MDA content (15.93%, 19.89%, and 24.47%), and soluble protein content (15.39%, 24.10% and 37.19%) all decreased. The soluble sugar content increased by 43.28% and 44.66% after MeJA25 and MeJA100 spraying, respectively.

### MeJA attenuates Cd toxicity by triggering the antioxidant defence system

**Figure 5:**
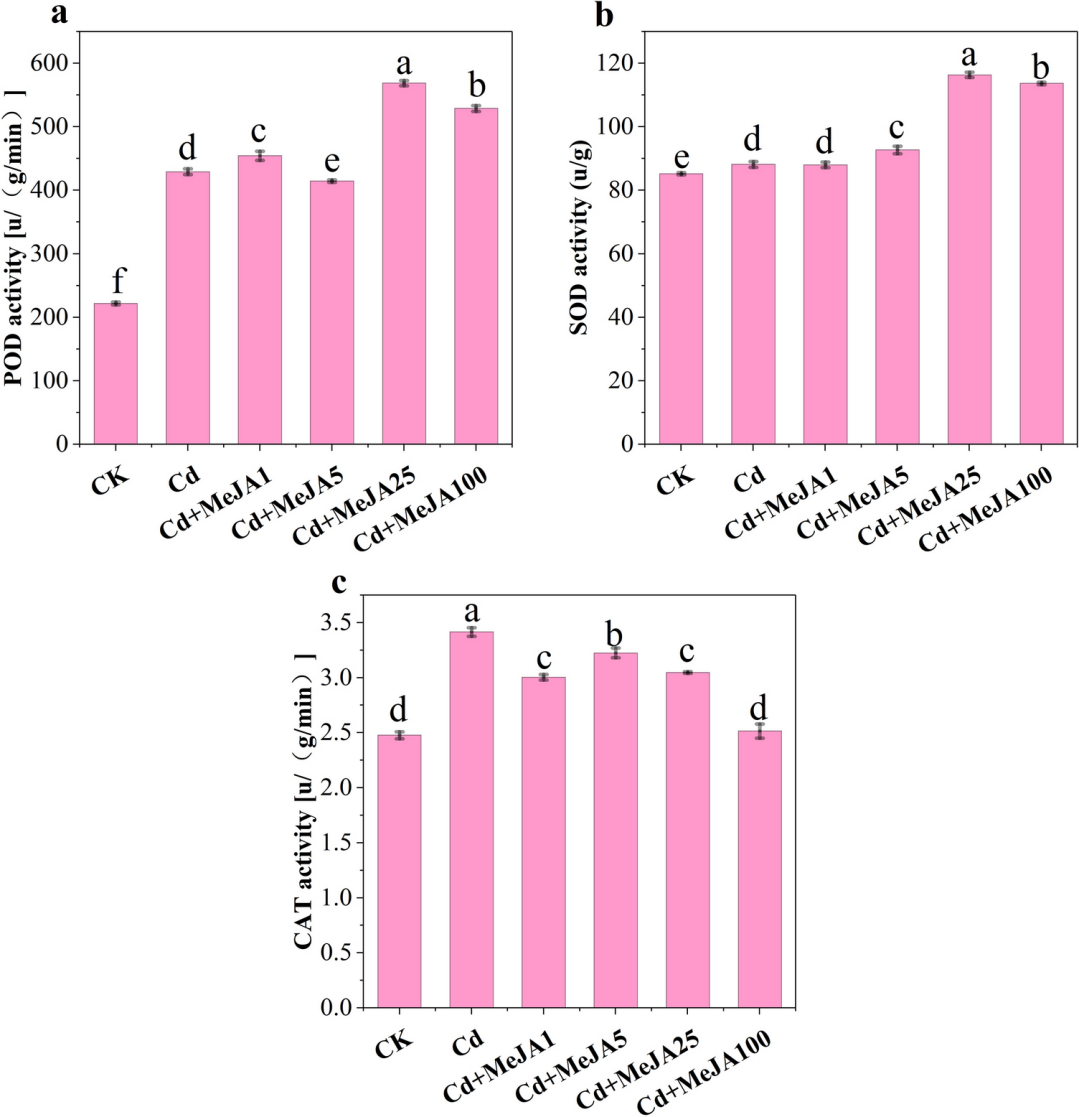
Effects of different concentrations of MeJA on POD, SOD and CAT activity in spinach. Note: (**A**) POD activity, (**B**) SOD activity, and (**C**) CAT activity.

**Figure 6:**
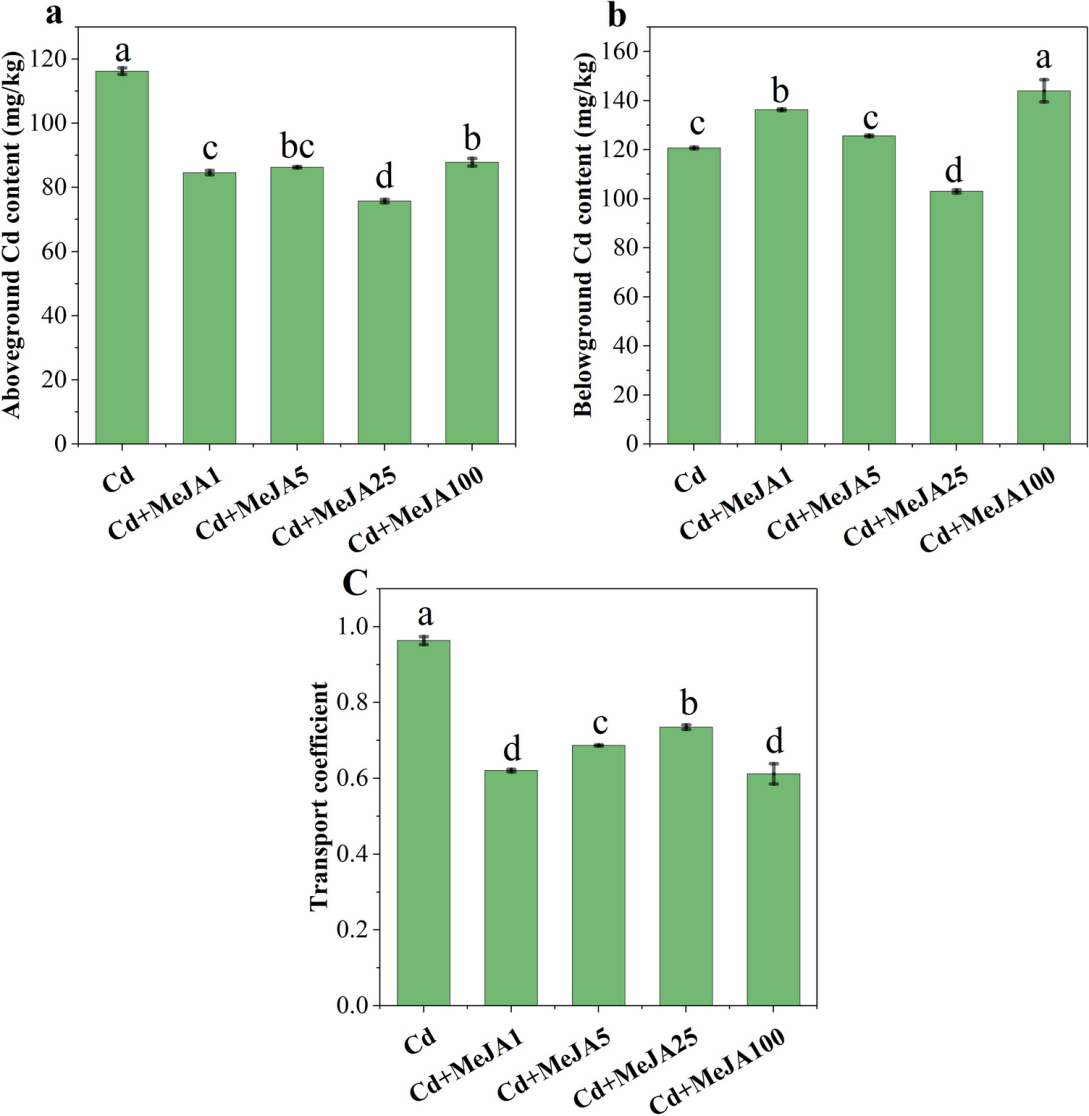
Effect of different concentrations of MeJA on the Cd content and transfer capacity of spinach. Note: (**A**) Aboveground Cd content, (**B**) belowground Cd content and (**C**) transport coefficient.

POD, SOD and CAT activities were significantly greater under Cd stress than under CK (Fig. 5, *p* < 0.05). The most pronounced increases in POD and SOD activity were observed after Cd stress with MeJA25 spraying (Fig. 6A,B); the increases were 32.49% and 31.99%, respectively. CAT activity decreased significantly, by 10.79% and 26.35%, after the application of MeJA25 and MeJA100, respectively.

### Foliar spraying of MeJA reduces Cd transport and accumulation in spinach

Figure 6 illustrates that different concentrations of MeJA inhibited the uptake of Cd in spinach to different degrees. Among them, the inhibitory effect of MeJA25 was greater, at 34.86% and 14.64% in the aboveground and belowground parts, respectively. The transport coefficient also reached a maximum value of 0.74 for MeJA25. Therefore, the reduction in Cd uptake and transport induced by MeJA spraying may be the underlying cause of MeJA-mediated alleviation of Cd toxicity.

### Correlation analysis of spinach assays under different concentrations of MeJA

**Figure 7:**
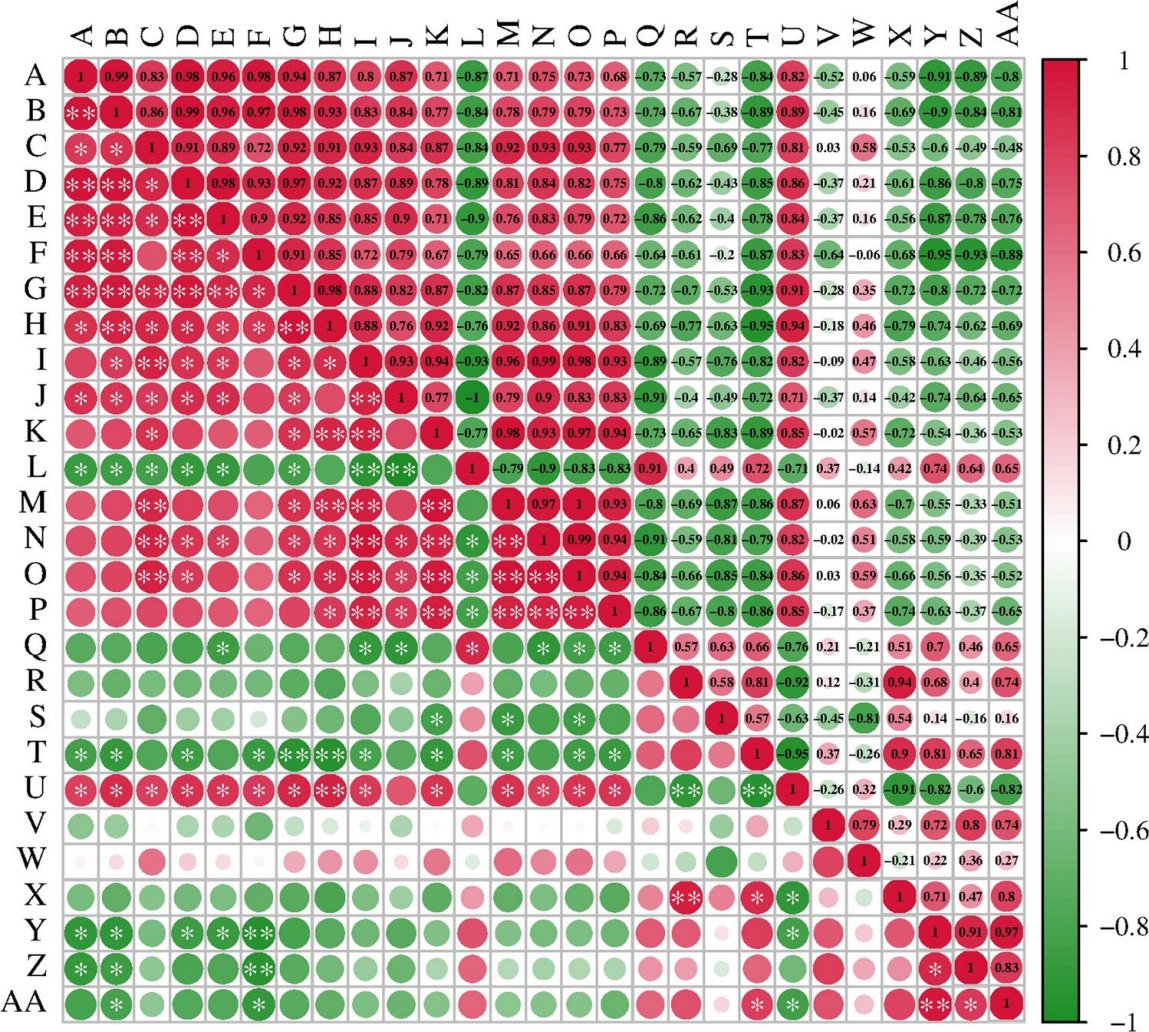
Correlation analysis plot of spinach under different concentrations of MeJA. Note: * indicates statistically significant (*p* < 0.05) differences between treatments according to Duncan’s multiple Range Test. A: Plant height; B: Root length; C: Stem thickness; D: Aboveground fresh weight; E: Aboveground dry weight; F: Belowground fresh weight; G: Belowground dry weight; H: Net photosynthetic rate (Pn); I: Stomatal conductance (Gs); J: Intercellular CO_2_ concentration (Ci); K: Transpiration rate (Tr); L: Stomatal limiting value (Ls); M: *Chl* a; N: *Chl* b; O: Total *chl*; P: Carotenoid content; Q: Electrical conductivity %; R: Free proline content; S: Malondialdehyde content (MDA); T: Soluble protein content; U: Soluble sugar content; V: POD activity; W: SOD activity; X: CAT activity; Y: Aboveground Cd content; Z: Belowground Cd content; AA: Transport coefficient.

The correlation coefficients (r) between different traits were estimated to be in the range of -0.95 to 0.99 with highly significant and significant values of 0.01 and 0.05, respectively (Fig. 7). Significant positive correlations (*p* < 0.05 or *p* < 0.01) were found between plant height, root length, stem thickness, aboveground fresh weight, aboveground dry weight, belowground fresh weight and belowground dry weight. However, the aboveground Cd content was significantly negatively correlated (*p* < 0.05 or *p* < 0.01) with plant height, root length, aboveground fresh weight, aboveground dry weight, belowground fresh weight and soluble sugar content. The translocation coefficient was significantly (*p* < 0.05) negatively correlated with root length, aboveground fresh weight and soluble sugar content, and significantly (*p* < 0.05) positively correlated with soluble protein content.

### Comprehensive evaluation of spinach growth under treatment with different concentrations of MeJA

A single index was used to evaluate the mechanism of spinach adaptation to different concentrations of MeJA in response to Cd treatment as described above. However, growth in the field was controlled by the combination of multiple factors, so the fuzzy mathematical affiliation function method was used to comprehensively evaluate 27 indexes, such as plant height, root length, stem thickness, aboveground fresh weight, aboveground dry weight, and belowground dry weight of spinach under the four treatments (Table 2). Based on the average value of the affiliation function, the ability to adapt to Cd stress under the four treatment levels decreased in the following order: MeJA25 > MeJA5 > MeJA100 > MeJA1.

**Table 2:** Comprehensive evaluation of spinach growth under different concentrations of MeJA.

Index	Under different concentrations of MeJA treatment			
	Cd + MeJA1	Cd + MeJA5	Cd + MeJA25	Cd + MeJA100
Plant height	0.0000	0.4674	1.0000	0.3967
Root length	0.0000	0.3415	1.0000	0.5305
Stem thickness	0.0000	0.3909	1.0000	0.5238
Aboveground fresh weight	0.0000	0.4107	1.0000	0.4120
Aboveground dry weight	0.0000	0.3398	1.0000	0.1359
Belowground fresh weight	0.0000	0.4675	1.0000	0.6250
Belowground dry weight	0.0001	0.3572	1.0000	0.7286
Net photosynthetic rate (Pn)	0.0000	0.3222	1.0000	0.9561
Stomatal conductance (Gs)	0.0000	0.8138	1.0000	0.7698
Intercellular CO_2_ concentration (Ci)	0.0000	1.0000	0.9211	0.3833
Transpiration rate (Tr)	0.0000	0.6232	0.7968	1.0000
Stomatal limiting value (Ls)	1.0000	0.0001	0.0790	0.6168
Chl a	0.0000	0.5884	0.9715	1.0000
Chl b	0.0000	0.7815	1.0000	0.7589
Total *chl*	0.0000	0.6650	1.0000	0.9381
Carotenoid content	0.0001	0.8374	0.6123	1.0000
Electrical conductivity %	1.0000	0.0000	0.0630	0.8727
Free proline content	0.3668	1.0000	0.3920	0.0000
Malondialdehyde content (MDA)	1.0000	0.5541	0.2970	0.0000
Soluble protein content	1.0000	0.6737	0.4044	0.0000
Soluble sugar content	0.0723	0.0001	0.7956	1.0000
POD activity	0.2593	0.0000	1.0000	0.7423
SOD activity	0.0000	0.1676	1.0000	0.9059
CAT activity	0.6890	1.0000	0.7495	0.0000
Aboveground Cd content	0.7308	0.8709	0.0000	1.0000
Belowground Cd content	0.8130	0.5528	0.0000	1.0000
Transport coefficient	0.0706	0.6067	1.0000	0.0001
Comprehensive evaluation of D value	− 0.5967	1.3155	2.4547	1.0992
Ranking	4	2	1	3

## Discussion

Cd is a highly toxic metal that hinders normal physiological and metabolic processes and disrupts plant functions^35^. In the present study, we found that Cd stress caused significant chlorosis in spinach leaves (Fig. 1B) and significantly inhibited the plant height, root length, stem thickness, aboveground biomass, and belowground biomass of spinach (Table 1). MeJA treatment enhanced the physiological parameters of spinach by increasing the uptake of valent cations and decreasing the Cd content and Cd^2+^ influx in spinach plants, similar to previous studies on pea^20^ and tomato^18^. Treatment with different concentrations of MeJA mitigated the effects of Cd stress on spinach to different degrees (Fig. 1C). In particular, the growth of spinach was well restored after after the plants were sprayed with MeJA25 in the Cd stress treatment (Fig. 1C and Table 1).

Cd stress usually causes oxidative stress in plants, due to the excessive production of reactive oxygen species (ROS, including H_2_O_2_, OH^−^ and O_2_^−^). These substances are toxic and highly reactive, oxidizing biomolecules such as proteins, carbohydrates, DNA and lipids^36^. The accumulation of proline, an important osmoregulator and effective scavenger of hydroxyl radicals, effectively protects plants from damage under adverse stress condition^37^. Both cell and organelle membranes can be oxidized by ROS. Peroxidation of polyunsaturated fatty acids in membranes leads to the production of MDA, which is therefore commonly used as a marker of lipid peroxidation and damage^38^. We found that Cd treatment significantly increased the conductivity, proline content, MDA content and soluble proteins content in spinach, whereas exogenous spraying with MeJA reduced these parameters to varying degrees (Fig. 4A–D). MeJA has the potential to overcome oxidative stress organelle and cell membranes^20^. Therefore, MeJA acted as a signalling molecule for proline and MDA, and induced Cd toxicity in spinach at the physiological level.

The activities of antioxidant enzymes and antioxidants are important indicators of cellular redox status^39^. Exogenous MeJA spraying at a certain level increased the activities of POD, SOD and CAT (Fig. 5A–C), possibly due to the increase in the activity of antioxidant enzymes involved in direct interactions with free radicals, such as superoxide, or increase in the bursting potential of cellular ROS through antioxidant enzymes to maintain redox homeostasis^40^. Additionally, Cd stress caused a significant reduction in the soluble sugar content of spinach. However, as the amount of MeJA applied increased, the soluble sugar content subsequently decreased. Exogenous MeJA spraying enhanced the tolerance of spinach to Cd, mainly because the increase in POD, SOD and CAT activity in response to oxidative stress alleviated the production of ROS, which in turn reduced the production of MDA, proline, and soluble protein in Cd-stressed plants^40^, thereby improving photosynthetic pigments and PSII efficiency^20^. Therefore, MeJA application effectively modulates lipid peroxidation and improves membrane stability by increasing the ROS scavenging potential of the antioxidant defence system.

The inhibition of plant growth by Cd stress leads directly to changes in the photosynthetic rate and photosynthetic pigment content, which in turn reduces the structural capacity and functional activity of PSII^20^. When Cd is taken up by plants, free Cd^2+^ ions disrupt cellular structures, and induce oxidative stress, leading to changes in cellular antioxidants that affect photosynthetic properties and ultimately transpiration and photosynthetic rates^9^. In the present study, Cd stress significantly suppressed Pn, Gs, Ci, and Tr in spinach (Fig. 2A–D), but Ls increased (Fig. 2E), indicating that the decrease in Pn was mainly caused by stomatal limitation^41^. In terms of the apparent photosynthetic indexes and chloroplast pigment expression, MeJA5, MeJA25, and MeJA100 spraying attenuated heavy-metal-induced photosynthetic damage (Figs. 2 and 3), and reduced Cd toxicity induced damage to the PSII machinery (Table 1). MeJA likely reduced the toxic effect of Cd on photosynthetic pigments and maintained the activity of PSII by decreasing the uptake of Cd into photosynthetic pigments, downregulating the electron transfer from PSII to PSI, increasing the photoprotective component of NPQ, and increasing the activity of some antioxidant enzymes^20,42^. JA has been found to attenuate heavy metal damage to photosynthetic organs and increase photosynthetic activity at both the structural and functional levels^43^. It has been reported that metal stress impedes the activities of various enzymes involved in the light-trapping complex and disrupts the Calvin cycle^44^. In the present study, the reduction in Pn was directly correlated with the reduction in pigment biosynthesis induced by Cd stress (Fig. 7). The entry of Cd into guard cells results in stomatal closure and reduced CO_2_ transport to chloroplasts, which leads to a reduction in Ci under Cd stress^43^. Cd stress inhibited the light responses of damaged chloroplasts. Cd altered the ultrastructure of chloroplasts and chlorophyll metabolism and decreased Gs and Tr^18^.The possible biochemical mechanism for the increase in photosynthetic pigments after MeJA treatment is likely to be the stimulatory effect on the activity of Calvin cycle enzymes and the enhancement of the uptake of minerals, particularly Mn, Mg, K, Ca and Fe, which are biochemicals involved in pigment biosynthetic reactions^45^. The recovery of chlorophyll and carotenoids after MeJA spraying following Cd stress (Fig. 3) may be attributed to the reduction in Cd accumulation and the regulation of ROS by antioxidant enzymes, and MeJA protects the chloroplast structure from Cd toxicity^20,46^. These results suggest that the exogenous application of MeJA effectively inhibits Cd release and maintains the metabolic activity of the plant, thereby protecting the photosynthetic mechanism.

MeJA can affect Cd transport and toxicity by changing the Cd accumulation pattern in the root system. Cd is taken up from the soil by plant roots and accumulates above and below ground. In our study, greater Cd accumulation was detected in the roots. According to Cataldo et al.^47^, Cd is mainly retained in plant roots, and only a relatively small amount is transported to the rest of the plant. MeJA-treated Cd-stressed spinach accumulated very low levels of Cd in the above and belowground parts, and MeJA25 significantly increased the transport coefficients of Cd to minimize Cd-induced damage, thereby promoting the overall growth of spinach (Fig. 6). The metal transporter proteins AtIRT and AtHMA4 reportedly to transport Cd as well as other divalent metal ions^48–50^. Li et al. showed that an exogenous supply of JA is an eco-friendly and effective method for reducing Cd accumulation in rice^48^. MeJA may have contributed to the decrease in root uptake of Cd by promoting the establishment of root morphology (Fig. 1A), promoting an increase in phenolic compounds in the root system, or facilitating the secretion of phenolic compounds from the root system into the nutrient solution^51^. At the same time, JA can mediate the biosynthesis of various organic acids in root secretions, or it may also release thiols that effectively chelate Cd in roots as a defence mechanism^52^. Therefore, a well-built root morphology may result in more selective permeability of the root cell membranes, leading to less Cd entering the root system and reducing the Cd uptake capacity of the root system. The present study provides an effective strategy for alleviating Cd stress in spinach via MeJA. However, molecular biology studies should further elucidate the potential mechanisms by which an exogenous supply of MeJA induces tolerance in plants under Cd stress.

## Conclusion

**Figure 8:**
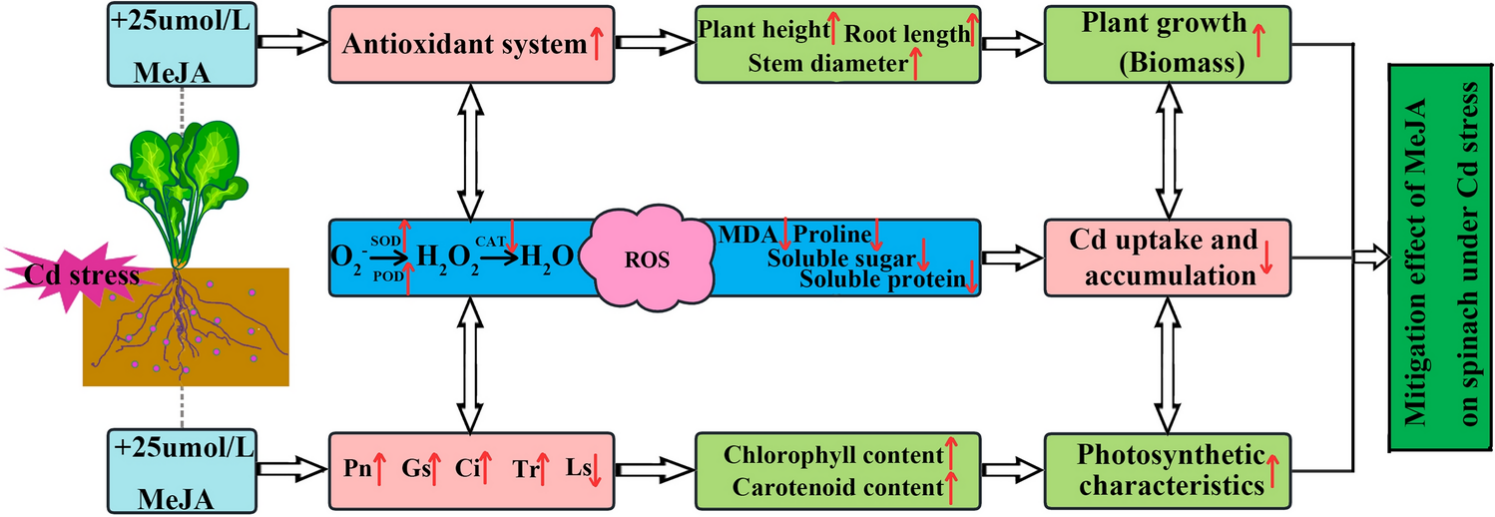
Model diagram of MeJA-mediated alleviation of Cd stress in spinach.

In the present study, an indoor experiment was conducted to determine how the exogenous application of MeJA alleviates Cd toxicity in spinach. Exogenous supplementation with MeJA increased the photosynthetic pigment content in spinach and inhibited the production of proline, MDA and soluble proteins mainly by increasing the gas exchange index in the plant and increasing the activities of POD, SOD and CAT to minimize the damage caused by Cd induction, thereby promoting the overall growth of spinach (Fig. 8). Taken together, these results have important practical implications, and MeJA improves the safety of vegetables by inhibiting the uptake and translocation of Cd into the edible portion of spinach grown on contaminated soil. Therefore, the application of MeJA25 can potentially reduce Cd uptake, decrease Cd toxicity, and increase yield in spinach under Cd stress conditions.

## Supplementary Information

Supplementary Table 1.

## Data Availability

All data generated or analysed during this study are included in this published article and its supplementary information files.
