# Supplementary material for: Methyl jasmonate alleviates Cd-induced lipid peroxidation in spinach by enhancing photosynthesis and the antioxidant defence system
Source: Sci Rep. 2025 Nov 18;15:40325. doi: 10.1038/s41598-024-60950-6 (PMC12627483; doi:10.1038/s41598-024-60950-6)
Supplement: Supplementary file 1 — Supplementary Table 1. [file 41598_2024_60950_MOESM1_ESM.docx]

**Methyl jasmonate alleviates Cd-induced lipid peroxidation in spinach**

**by enhancing photosynthesis and the antioxidant defence system**

Mingjun Miao^1,2,4^, Jiajia Li^1^, Xiaokui Lei^1,2^, Jichao Liao^1^, Jian Zhong^1,2^,

Ju Li^1,2,4^, Zhi Li^1,2,4^, Liang Yang^1,2,4^, Yanqin Ma^1,2^, Yuejian Li^1,2,4^, Fengshan Li^5^, Wei chang^2,3,4^

Correspondence: Wei chang: [471243498@qq.com](mailto:471243498@qq.com)

Tel.: 028-84590536

^1^Horticulture Research Institute, Sichuan Academy of Agricultural Sciences, Vegetable Germplasm Innovation and Variety Improvement Key Laboratory of Sichuan Province, Chengdu 610066, China.

^2^Horticultural Crops Biology and Germplasm Enhancement in Southwest Regions Key Laboratory of Ministry of Agriculture and Rural Affairs, Chengdu 610066, China.

^3^Institute of Sichuan Edible Fungi, Chengdu 610066, China.

^4^Sichuan Vegetable Engineering Technology Research Center, Chengdu 610066, China.

^5^Pengzhou Rural Investment Development Co., Pengzhou 610000, China.

**Supplementary Table1 Different treatment combinations**

| Treatments | Reagent composition in different treatments |
| --- | --- |
| CK | 0 μmol/L CdCl_2_, 0 μmol/L MeJA |
| Cd | 50 μmol/L CdCl_2_, 0 μmol/L MeJA |
| Cd+MeJA1 | 50 μmol/L CdCl_2_, 1 μmol/L MeJA |
| Cd+MeJA5 | 50 μmol/L CdCl_2_, 5 μmol/L MeJA |
| Cd+MeJA25 | 50 μmol/L CdCl_2_, 25 μmol/L MeJA |
| Cd+MeJA100 | 50 μmol/L CdCl_2_, 100 μmol/L MeJA |
